# Network Analyses Reveal Pervasive Functional Regulation Between Proteases in the Human Protease Web

**DOI:** 10.1371/journal.pbio.1001869

**Published:** 2014-05-27

**Authors:** Nikolaus Fortelny, Jennifer H. Cox, Reinhild Kappelhoff, Amanda E. Starr, Philipp F. Lange, Paul Pavlidis, Christopher M. Overall

**Affiliations:** 1 Department of Biochemistry and Molecular Biology, University of British Columbia, Vancouver, British Columbia, Canada; 2 Centre for Blood Research, University of British Columbia, Vancouver, British Columbia, Canada; 3 Department of Oral Biological and Medical Sciences, University of British Columbia, Vancouver, British Columbia, Canada; 4 Department of Psychiatry, University of British Columbia, Vancouver, British Columbia, Canada; 5 Centre for High Throughput Biology, University of British Columbia, Vancouver, British Columbia, Canada; Yale University, United States of America

## Abstract

Proteolytic processing is an irreversible posttranslational modification affecting a large portion of the proteome. Protease-cleaved mediators frequently exhibit altered activity, and biological pathways are often regulated by proteolytic processing. Many of these mechanisms have not been appreciated as being protease-dependent, and the potential in unraveling a complex new dimension of biological control is increasingly recognized. Proteases are currently believed to act individually or in isolated cascades. However, conclusive but scattered biochemical evidence indicates broader regulation of proteases by protease and inhibitor interactions. Therefore, to systematically study such interactions, we assembled curated protease cleavage and inhibition data into a global, computational representation, termed the protease web. This revealed that proteases pervasively influence the activity of other proteases directly or by cleaving intermediate proteases or protease inhibitors. The protease web spans four classes of proteases and inhibitors and so links both recently and classically described protease groups and cascades, which can no longer be viewed as operating in isolation *in vivo*. We demonstrated that this observation, termed reachability, is robust to alterations in the data and will only increase in the future as additional data are added. We further show how subnetworks of the web are operational in 23 different tissues reflecting different phenotypes. We applied our network to develop novel insights into biologically relevant protease interactions using cell-specific proteases of the polymorphonuclear leukocyte as a system. Predictions from the protease web on the activity of matrix metalloproteinase 8 (MMP8) and neutrophil elastase being linked by an inactivating cleavage of serpinA1 by MMP8 were validated and explain perplexing *Mmp8* ^−/−^ versus wild-type polymorphonuclear chemokine cleavages *in vivo*. Our findings supply systematically derived and validated evidence for the existence of the protease web, a network that affects the activity of most proteases and thereby influences the functional state of the proteome and cell activity.

## Abstract 2

Network modeling of interactions between proteases and their inhibitors reveals a network of new protein connections and cascades in the protease web.

## Author Summary

Proteases modify the structure and activity of all proteins by peptide bond hydrolysis and are increasingly recognized as integral regulatory components of numerous biological mechanisms. Deregulated protease activity is a common characteristic of many diseases. However, protease drug development is complicated by an incomplete understanding of protease biology. One missing piece in this puzzle is the interplay between proteases: Some proteases activate other proteases, whereas some proteases inactivate inhibitors, leading to currently unpredictable cleavage of additional proteins. Using database annotations we mathematically modeled protease interactions. Our model includes 1,230 proteins and shows connections between 141,523 pairs of proteases, substrates, and inhibitors. Thus, proteases interact on a large scale to form the protease web, which links most studied groups of proteases and their inhibitors, indicating that the potential of regulation through this network is very large. We found that this interplay is robust to targeted or untargeted pruning of the protease web and that protease inhibitors are central to network connectivity. Our model was used to decipher proteolytic pathways that drive inflammatory processes *in vivo*. Consequently, protease regulatory interactions should be recognized and explored further to understand *in vivo* roles and to select better drug targets that avoid side effects arising from inhibition of unexpected activities.

## Introduction

Proteolysis, the hydrolysis of peptide and isopeptide bonds in protein substrates by proteases (also termed peptidases or proteinases [1]), affects every protein at some point during its lifetime. The outcomes of proteolysis are of two kinds: Protein degradation ablates protein function by breakdown to amino acids, whereas proteolytic processing is an irreversible posttranslational modification to precisely produce modified, stable protein chains. The length of this cleavage product is defined by the substrate site specificity of the protease catalyzing the reaction, which can be exquisite. Processed proteins often have radically altered activity, protein interactions, structure, or cellular location and hence are implicated in many human diseases [2]–[4]. Recent research has focused on identifying the cleavage products of protease activity in cell culture and *in vivo* as a means of understanding their biological roles and hence guiding drug target identification and validation [5]. This need has led to the development of genomics and proteomics approaches that have come to be termed degradomics [6],[7] in which the specialized subfield known as terminomics that identifies N termini [8]–[10] and C termini [11],[12] has seen recent rapid development. In one such terminomics analysis of murine skin *in vivo*, ∼44% of identified N termini mapped to internal positions in proteins, revealing proteolytic cleavage after translation as part of protein maturation and function [13]. With ∼68% of identified N-termini being internal, human erythrocytes have been found to possess an even higher proportion of processed proteins [14]. These recent findings demonstrate that proteolytic processing is a widespread and functionally important posttranslational modification. Thereby, proteolytic processing modifies the activity of many more proteins than currently appreciated from conventional shotgun proteomics analyses and biological studies.

As exemplified by N-terminal cleavage of chemokines [6], the activity of a protein often depends on the exact position and nature of its N and C termini [15]. Therefore, identifying the termini of proteins is essential for functional insight into protein bioactivity, annotation of proteins in the Human Proteome Project, and drug development [14]. However, deeper biological insight requires identifying the protease responsible for generation of neo-termini that distinguish cleavage products from the original protein termini. Whereas low- and high-throughput methods to identify the *in vitro* substrate repertoire of proteases, also known as the substrate degradome [7], are well established, *in vivo* identification is problematic [16]. *In vitro* experiments can only indicate potential cleavage *in vivo* because of difficulties assigning precise parameters governing cleavage in the actual biological system, such as protease and substrate colocalization spatially and temporally, presence of inhibitors, zymogen activation, pH, ion concentrations, interaction with nonprotein compounds [17], as well as O-glycosylation or phosphorylation of the protease or substrate [18]. Hence, posttranslational modifications of proteases, inhibitors, and their substrates add complexity to the dynamic nature of the proteome and cell responses. Thus, an observed cleavage *in vitro* might not occur *in vivo*—that is, “just because it can (*in vitro*) does not mean it does (*in vivo*)” [5].

*In vivo* studies, which rely on comparing samples of protease knockout or inhibition to controls, are hampered in particular because the underlying biological system reacts to the removal of a protease or inhibitor in complex and unpredictable ways. For example, a protease knockout can lead to alterations in gene expression profiles of proteases, inhibitors, and substrates [13],[19], due to the biological consequences of altered substrate cleavages *in vivo*, including cleavage of transcription factors [20]. Another factor is the activation of other proteases in the system through increasingly recognized activation cascades of protease zymogens by other proteases and the proteolytic regulation of protease inhibitor activity by nontarget proteases that cleave and inactivate the inhibitor. For example, serpins and cystatins inhibit serine and cysteine proteases, respectively, but when cleaved by a matrix metalloproteinase (MMP), the inhibitor is inactivated and the protease remains active [13],[21]–[23]. Through activating and inactivating cleavages of other proteases and inhibitors, a protease thereby indirectly influences the activity of additional proteases. Such interactions can lead to knock-on effects that alter the cleavage of a range of additional protein substrates that are not direct substrates of the protease. Furthermore, titration of inhibitors upon covalent or tight interaction with one protease can reduce the availability of free inhibitors to regulate other proteases. Consequently, phenotyping protease and inhibitor genetic knockout mice is complicated, which also hampers biological understanding and drug target validation of proteases.

Protease biology is also complex due to the large protease numbers in humans (460) and mice (525), which form the second largest enzyme family after ubiquitin ligases in these organisms [24]. Moreover, an additional 93 and 103 are predicted to be inactive proteases in human and mouse, respectively, which often can function as dominant negative counterparts [24]. Protease numbers are almost equally distributed in the intracellular and extracellular environments, and other than some proteases that segue between these two compartments, this distribution partitions and limits their potential interactions with each other. In an effort to systematically comprehend this complex biology, proteases are grouped by the MEROPS database, which is assembled from biochemical experimental data curated from the literature, into seven classes, five of which are found in human and mouse, according to the active site residue catalyzing substrate cleavage, and into clans based on the structure of the active site [25]. Similarly, inhibitors are commonly grouped according to the class of proteases they inhibit, with several inhibitors exhibiting broad inhibitory activity against proteases from more than one class. Interactions between proteases of the same class are well established as part of classically described cascades of proteases such as the complement [26]–[28] and coagulation [29],[30] systems, and newer recognized cascades such as kallikreins [31] and caspases in apoptosis [2],[32]–[34]. However, wide-ranging additional protease interactions have also been proposed to extend more globally to link networks forming what was termed the protease web [35]. The protease web was defined as the universe of cleavage and inhibition interactions between proteases and their inhibitors. Stemming from examples in simple systems such as *in vitro* biochemical analyses and early *in vitro* and cell culture degradomics analyses of protease substrates [36]–[38], and mRNA transcript analyses in cancer upon administration of protease inhibitors or tissue inhibitor of metalloproteinase (TIMP) overexpression and knockout studies [19], the protease web concept has been well supported. Extending terminomics analyses to *in vivo* situations, for example skin inflammation in wild-type versus *Mmp2* knockout mice *in vivo*, has revealed hitherto biologically relevant and unsuspected critical connections of MMPs in regulating the complement and coagulation cascades and the plasma kallikrein system, which regulates vessel permeability through bradykinin excision and release from kininogen [13].

Such interactions between protease families were shown to create small networks in specific cases [13],[19],[39],[40], but the full extent of the protease web, the fraction of proteases and inhibitors involved, and hence the regulatory potential of this network remain underexplored and underappreciated despite the potentially wide impact on the functional state of proteomes. Furthermore, the protease web is a black box with an unknown mechanism of regulation—it is unclear whether it follows a super structure of known cascades, where signals are amplified downstream, or forms more of a network, where signals can flow in multiple directions with multiple positive and negative feedback loops [35],[40]. Similarly, it is unclear which are the main regulatory protein switches controlling subparts of the network. Descriptions of the protease web are difficult to assemble, as many proteases remain poorly studied and characterized. Likewise, many proteases have no described inhibitors and many predicted inhibitors have unknown protease targets and deorphanization examples are uncommon [41].

Here, we assessed the global extent and structure of protease interactions computationally. Graph models are used to describe multiple interactions between many elements and have been applied extensively in research on various biological networks. We represented existing biochemically validated data on protease cleavages and inhibition as annotated in the manually curated database TopFIND [42] as organism-specific networks. TopFIND stores established biochemical information on substrate cleavage and protease inhibition from MEROPS [25], the most complete collection of such data, most of it published, and combines it with published high-throughput terminomics and degradomics datasets as well as protein annotations from UniProt [43] for five different organisms. Our analyses revealed a large and pervasive network spanning all known cascades and four of the five protease classes present in human and mouse tissues. The network is highly connected in that via a few connections a protease can potentially influence many other proteases, with inhibitors often taking a special role as key connectors in the protease web. We demonstrate the utility of our analysis by applying the network to gain mechanistic *in vivo* insights into protease web effects, which we then validated *in vitro*, in cell culture, and *in vivo*.

## Results

### Protease Web Data

**Figure 1:**
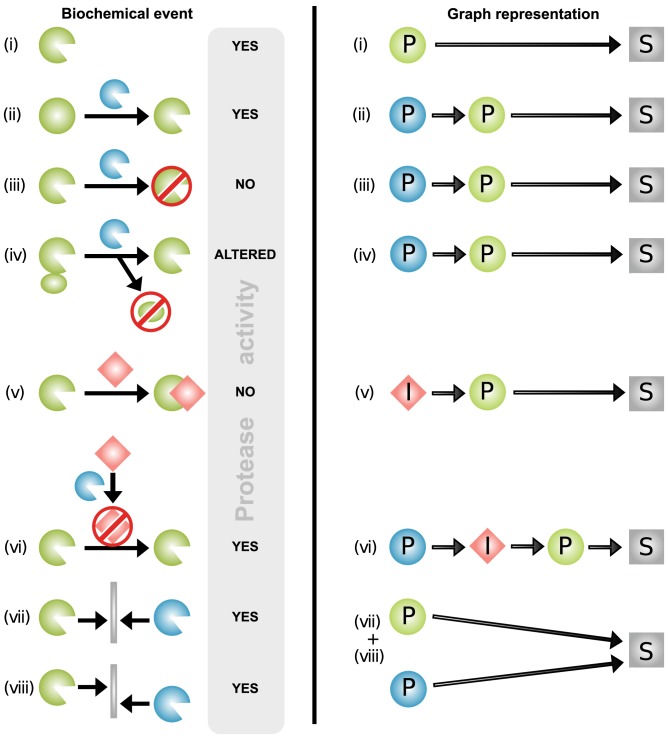
Biochemical protease interactions represented by graph theory. Proteases influence cleavage of substrates both directly and also indirectly through cleavage of other proteases and inhibitors. Protease interactions as represented biochemically (left) and by graph theory (right). Proteases are green or blue, inhibitors are red, and other substrates are grey. Examples of protease interactions (cleavage and inhibition events) are outlined on the left: (i) In the simplest case, a protease directly cleaves a substrate, as indicated by the presence of proteolytic activity, with no further interactions. A protease can also indirectly influence cleavages by cleaving another protease for (ii) zymogen activation [76], (iii) catalytic domain removal, or (iv) exosite domain removal [77]. This will increase (ii), decrease (iii), or alter (iv) [78] the activity of the affected protease and thereby influence the cleavage of its substrates. (v) If a protease inhibitor is present, the protease does not cleave substrates. (vi) An inhibitor can be cleaved and inactivated by another protease [13],[79], which leads to increased cleavage of substrates by its cognate protease. Proteases also compensate for loss of function of other proteases or complement their activity by (vii) cleaving the same substrate at the same site or (viii) substrate cleavage by one protease can depend on prior cleavage by another protease at a different site. By graph theory of protease interactions (right), all proteins are nodes. Proteases (P) are represented as green or blue circles, inhibitors (I) as red diamond shapes, and substrates (S) are grey squares or rectangles. An edge from protein A to protein B signifies a direct regulatory influence from A on B. Such a regulatory effect could either be a cleavage or inhibition, resulting in higher, lower, altered, or unchanged activity of the target.

Functional protease interactions comprising cleavage and inhibition events influence the *in vivo* cleavage of substrates in many ways. Cleavage of a substrate by a protease is a direct event, and as shown in Figure 1, by cleaving other proteases and protease inhibitors, one protease can activate, inactivate, or alter the activity of a second protease, thereby indirectly influencing the cleavage of substrates of another protease. To assess the global extent of such effects, we represented protease interactions as a graph, connecting proteases and protease inhibitors to their established substrates and protease targets, respectively. The resulting graph contains nodes, which are proteins, and edges, which represent cleavages or inhibitions. Edges link proteases to their substrates and protease inhibitors to their target proteases. Therefore, edges are directed: an edge from protein X to protein Y signifies cleavage or inhibition of Y by X but does not contain information about cleavage or inhibition of X by Y. In graph theory, the latter would require another edge with the opposite directionality. Figure 1 outlines functional protease interactions and how they are represented in small graph models, which were then aggregated to represent the full complexity of the protease web based on curated biochemical data as described below.

As input to our analysis of the protease network, we used the TopFIND v 2.0 knowledgebase [44] to retrieve validated cleavage and inhibition data mostly annotated from published experiments. TopFIND contained 4,774 cleavages for *Homo sapiens*, 3,679 for *Mus musculus*, 426 for *Escherichia coli*, 190 for yeast, and 43 for *Arabidopsis thaliana*. Due to the low number of cleavages annotated for other organisms, we focused our analysis on human and mouse. Only proteins performing an annotated cleavage or inhibition were added, and then these were connected via edges representing the biochemical reactions as explained in Figure 1. These networks extend the protease web, which contains only proteases and inhibitors, by also including all other substrates of proteases, and hence represent the annotated functional proteolytic interactions between the substrates in the proteome and the protease web. The human and murine networks (with 1,230 and 1,393 nodes, respectively) are shown in high resolution upon click-to-zoom in Figure S1 and available for download as a Cytoscape file, gml file, and R objects at www.chibi.ubc.ca/ProteaseWeb and http://clipserve.clip.ubc.ca/supplements/protease-web.

The human and murine proteolytic networks show that the majority of proteins are connected and only very few are in unconnected components. Thus, in both networks, the Largest Connected Component (i.e., the biggest group of nodes directly or indirectly connected) encompasses the vast majority of these proteins—1,183 of 1,230 (96%) in human and 1,377 of 1,393 (99%) in mouse (Table 1). This remarkable connectivity is particularly surprising given the incompleteness of annotation currently available in the databases. Indeed, Table 1 shows that of 460 human proteases, only 244 (53%) have one or more known and annotated substrates. In mouse this number is even lower, with only 88 of 525 (17%) proteases having a substrate annotated. Furthermore, even the data on these proteases are incomplete and biased, with most substrates assigned to few, well-studied proteases. Figure S2 shows the out-degree (i.e., the sum of cleavages catalyzed by a protease or the sum of inhibitions caused by a protease inhibitor) for proteases and inhibitors having any annotated cleavage or inhibition, respectively. Although few proteases have a large known substrate repertoire (higher out-degree), most proteases have very few known substrates. Although this could be due to high substrate specificity, it is more likely that these proteases simply received less attention in studies dedicated to discover substrate repertoires. This effect is especially pronounced for the mouse data, where 80% of total cleavages (2,938 of 3,679) are assigned to three proteases—cathepsin D (UniProt: P18242), cathepsin E (UniProt: P70269), and MMP2 (UniProt: P33434)—and are mostly derived from high-throughput proteomics screens. Accordingly, the annotations differ strongly between human and mouse. Although the networks have similar size (1,230 and 1,393 nodes, respectively), they overlap minimally, with only 126 of 3,852 connections in mouse (3.3%) reflected in 122 of 4,905 human connections (2.5%). However, we suggest that the small overlap is mostly due to differences in the state of data annotation between the networks rather than to actual differences in the evolution of these networks.

**Table 1:** Human and mouse proteolytic networks created from all annotated proteases, inhibitors, and substrates.

Organism	Total Nodes	Largest Connected Component	Proteases with Substrates^a^	Inhibitors with Target Proteases^a^	Edges
Human	1,230	1,183	244	41	4,905
Mouse	1,393	1,377	88	47	3,852

a Protease and inhibitor numbers refer to those with at least one annotated cleavage or inhibition, respectively, in MEROPS and TopFIND.

The human data are further biased in that proteases and inhibitors are largely overrepresented as substrates themselves (Figure S3). Strong representation of protease–protease cleavages is expected because many proteases are synthesized as zymogens requiring proteolytic cleavage for activation by other proteases. Indeed, this strong enrichment is found in the human TopFIND/MEROPS data, but less so in mouse. We compared these values to a terminomics data set of cleavages in mouse skin [13], which more accurately reflects reality because terminomics analyzes N termini in an unbiased fashion. However, in this *in vivo* data set, inhibitors, and not proteases, were overrepresented as processed proteins, indicating that the overrepresentation of proteases as cleavage substrates in the human *in vitro* database is likely exaggerated.

The observed data biases likely resulted from the nature of biochemical studies, where many substrates were identified for some “interesting” proteases (target bias) and “interesting” proteins are more likely to be tested as substrates (substrate bias). Substrate bias is especially found for proteases themselves, which are preferably tested as substrates in zymogen activation studies. With the advent of degradomics utilizing proteomics methods dedicated to substrate discovery, we anticipate both an increase in target bias in the future with many substrates identified for a few proteases, and a decrease in substrate bias where any protein can be identified as a substrate without prior selection of interesting candidates. Therefore, the cleavages annotated represent a biased fraction of the biochemically possible cleavages in the organism compared with an unknown number of as yet uncharacterized cleavages. On these grounds, the high connectivity in both the mouse and human networks is even more noteworthy because future information can only further increase connectivity. The observed, extensive interactions between proteases and inhibitors are further characterized as described in the following.

### Protease Web Structure

**Figure 2:**
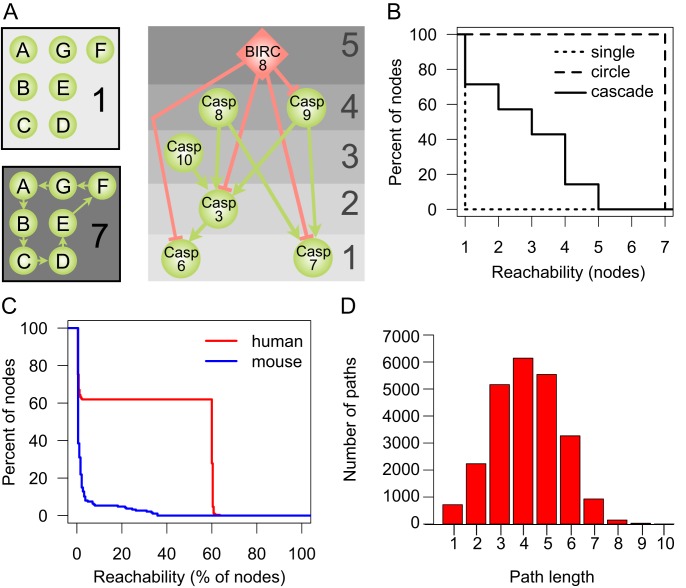
Reachability in network examples and the human and murine protease webs. Connectivity in the protease networks as measured by the reachability distribution of nodes in the network. (A) Reachability in three theoretical model networks: In an unconnected network without edges, each node has a reachability of 1. In a strongly connected network, where each node can reach each other node, the reachability of each node is the sum of nodes. In a hierarchical, cascade-like network (apoptosis cascade taken from KEGG [80]), reachability values are high for upstream regulators and decrease as one descends the cascade towards the downstream effector proteins. For each protein, the corresponding reachability values are shown on the right. Proteases are represented as green circles and inhibitors as red diamonds. Edges are cleavages (green, with arrow head) and inhibitions (red, with “T” head). Although these two types of edges have biologically distinct interpretations, the implication for the graph model and reachability is identical. (B) Reachability values of nodes in a theoretical hierarchical cascade (cascade), unconnected (single), or strongly connected (circle) networks shown in (A). Reachability is plotted as an inverse cumulative function of the percentage of nodes, which can reach a given minimum number of nodes in the corresponding network. (C) Inverse cumulative function of reachability values of the largest connected components of the human protease web (255 nodes, red line) and the mouse protease web (187 nodes, blue line). Reachability is plotted as the inverse cumulative function of the percentage of nodes that can reach a given minimum percentage of nodes in the corresponding network. (D) Histogram of the path length of all shortest paths in the human network comprised of a total of 24,166 paths.

In the interactions between proteases in proteolytic signaling pathways, there are major upstream regulators or initiation factors, whose proteolytic activity leads to the cleavage of downstream proteases, which in turn activate even further downstream factors that finally cleave and activate the effector molecules at the end of the pathway. A special case of proteolytic pathways are activation cascades, where signal amplification occurs to generate large quantities of the end protein products in seconds as classically described for coagulation [29],[30]. To investigate whether the connections in the overarching protease web follow such a pathway or cascade (hierarchical) structure, we used a graph measure termed reachability. Reachability of node X denotes the number of other nodes Y where there is a path from X to Y in the network. A path is a sequence of directed edges connecting X and Y, following the directionality of edges in the network. The path from X to Y can therefore be different from the path from Y to X (and the existence of one does not guarantee the existence of the other). In the protease web, reachability corresponds to the number of proteins that can be influenced by one protease or inhibitor. Figure 2A outlines reachability values of nodes in three theoretical examples: (i) an unconnected (single), (ii) a strongly connected (circle), and (iii) a cascade-like network (cascade). Figure 2B shows the respective distribution of reachability values of these three theoretical examples.

We next compared the theoretical reachability distributions with the distributions observed in our human and mouse protease networks. In order to specifically describe the selective connectivity between proteases and inhibitors, which form the protease web, we excluded from further analysis other simple substrates (nonprotease and noninhibitor proteins), whose reachability in the network is 1 by definition. Table 2 summarizes the resulting protease web networks for human (340) and mouse (220) proteins that have annotated cleavages or inhibitions. In analyzing the human and mouse protease webs, we further identified one dominant “largest connected component” comprised of 255 proteins for human and 187 proteins for mouse. Figure 2C compares the distribution of reachability scores in the largest connected component in mouse (blue curve) and human (red curve). In mouse, reachability indicates a cascade-like, hierarchical network, where most nodes have a very low reachability and fewer nodes have gradually higher reachability. In contrast, the reachability distribution of the human network follows a strongly bimodal distribution: 158 (62%) nodes reach 153 (60%) or more nodes. This is very high reachability that is most similar to the circle graph in Figure 2B, where any node can reach any other node. For a biological system, this implies that 158 proteases or inhibitors have the potential to regulate the activity of 153 or more other proteases and inhibitors in the network. In other words, there are one or more directed paths between 24,166 pairs of proteases in the human protease web, which are 37% of all 64,770 possible directed connections between pairs of 255 proteins. This number of connections between pairs rises to 141,523 paths when substrates are added (network with 1,230 nodes). This highlights the high degree of connectivity between proteases and inhibitors. Reachability between nodes does not take the path length between nodes into account and so might be the result of very long and hence biologically irrelevant paths in the network. However, this possibility can be excluded as most paths have a length of just four (Figure 2D). The lack of connectivity in the mouse network is not surprising given the small overlap between the two networks. We assume that this difference is due to data biases rather than a real biological difference, and accordingly we focused on characterizing the extensive and more complete human network.

**Table 2:** Proteins comprising the human and mouse protease webs.

Organism	Proteins with MEROPS ID	Protease Web^a^		Largest Connected Component			
		Nodes	Edges	Nodes	Proteases^b^	Inhibitors^b^	Edges
Human	755	340	1,264	255	215	40	1,238
Mouse	696	220	415	187	141	46	404

a Only nodes having a MEROPS ID and are part of a cleavage or inhibition are in the protease web.

b Proteases and inhibitors are assigned based on the MEROPS IDs of the proteins.

**Figure 3:**
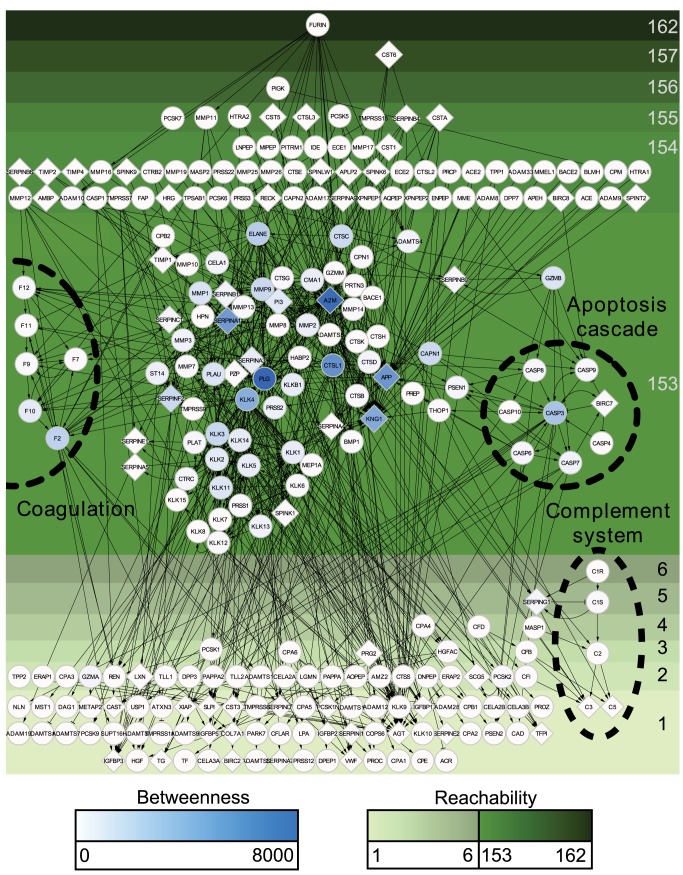
The largest connected component of the human protease web. The structure of the core of the human protease web is comprised of 255 connected proteases and inhibitors that form the largest connected component. Proteins are designated by their UniProt gene names. Proteases are circles and inhibitors are diamonds. Nodes are color-shaded according to their betweenness. All nodes are positioned from top to bottom by decreasing reachability, which is indicated by the depth of shade of the green background. Edges are cleavages (with arrow head) or inhibitions (“T” head). Nodes of known protease cascades are labeled and marked by dashed circles. The figure is rendered in high resolution for click to zoom.

High connectivity in the human protease web is due to a strongly connected component (87 nodes), a subgroup of nodes within the largest connected component, that can directly or indirectly reach each other and hence have the same reachability value of 153 (Table 3). We visualized this effect in Figure 3, where nodes of the human protease web are shown separated by their reachability. Upstream of the strongly connected component are 71 nodes with reachability higher than 153; these nodes can reach the strongly connected component, but cannot be reached from it. Downstream (with reachability smaller than 7) are 97 nodes, which cannot reach the strongly connected component. The nodes in Figure 3 are also colored according to their centrality in the network, as measured by node betweenness [45]. Betweenness is calculated by first finding the shortest paths (as explained above) between all 64,770 pairs of nodes in the network and then counting the number of times a node appears in these paths. Notably, all nodes with high betweenness are found in the strongly connected component; these nodes tether the network together. Nodes with high betweenness or reachability are listed in Table S1.

**Table 3:** Reachability values of nodes in the human protease web.

Number of Nodes	Reachability
97	<7
87	153
71	>153

**Figure 4:**
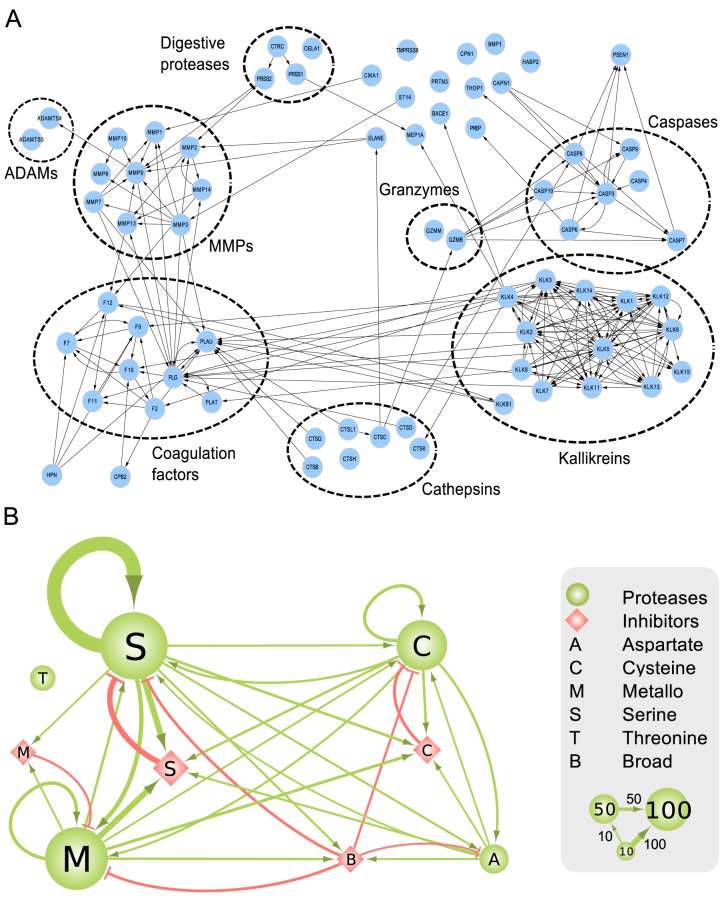
Interactions between protease groups in the human protease web. (A) Click-to-zoom figure of detailed connections between pathways and protease groups in the strongly connected component of the network. The network presented is limited to proteases (no inhibitors) with a reachability of 153 from Figure 3. Nodes are proteases and edges are cleavages. Proteases are designated by their UniProt gene names. (B) Interactions between classes of proteases and their inhibitors. Nodes are classes of proteins: classes of proteases are green circles; classes of protease inhibitors are red diamonds. The size of the nodes represents the number of proteins in each class as exemplified with groups of 10, 50, and 100 nodes in the legend. Protein classification: “M” are metallo, “S” are serine, “C” are cysteine, “A” are aspartate, and “T” are threonine proteases (as classified in MEROPS) or the corresponding inhibitors (as annotated in neXtProt). “B” are broad-spectrum inhibitors that are annotated to inhibit more than one class of protease and include A2M, serpin B4, serpin B9, PZP, histidine-rich glycoprotein, ovostatin homolog 1, and reversion-inducing cysteine-rich protein with Kazal motifs. Edges are cleavages (green, with arrow head) or inhibitions (red, with “T” head). Thickness of edges corresponds to the number of cleavages or inhibitions between the classes as exemplified with edges corresponding to 10, 50, or 100 interactions in the legend.

Figure 3 shows that our network data from MEROPS/TopFIND contain all the known proteolytic pathways (e.g., coagulation, complement system, apoptosis, and kallikreins) as they were discovered, published, and annotated previously in MEROPS (detailed in Figure S4). In addition, these proteolytic pathways are extended by connections linking known pathways with other pathways and additional proteases. Details of these connections can be found in Figure 4A, which shows separated protease groups in the strongly connected component after removing inhibitors. Figures 3, 4A, and S4 show that the observed connectivity in the protease web is caused by the concerted action of defined protease cascades and key protease inhibitors: alpha-2-macroglobulin (A2M, UniProt: P01023), amyloid precursor protein (APP, UniProt: P05067), kininogen 1 (KNG1, UniProt: P01042), and alpha-1-antitrypsin (also known as serpin A1) (SERPINA1, UniProt: P01009). Whereas intragroup connections are pervasive as expected, intergroup connections are also considerable, in particular between coagulation factors and kallikreins or MMPs, but also including cathepsins and caspases. These findings are confirmed in Figure 4B, which shows that connections among four of the five classes of proteases and protease inhibitors in human are extensive. Importantly, Figure 4B also shows proteases frequently cleaving inhibitors of other protease classes, an important regulatory aspect of protease activity. Only threonine proteases, which are found exclusively in large specialized cell organelles termed the proteasome and immunoproteasome, remain isolated from connections with other proteases and inhibitors according to current data. Note added in proof: However, a recent publication shows that the threonine proteasomal proteases are cleaved by intracellular MMP-12. Thus, all five classes of proteases in human and mouse are interconnected [81].

### Theoretical Network Analysis of the Protease Web

From a biological standpoint, the highly interconnected (reachable) nature of the protease web was surprising and underappreciated in the literature. To explore the degree to which this result is statistically surprising given the properties of the proteins making up the network, we investigated theoretical network models as well as randomized versions of the network. We first compared the protease web to two commonly used generative network models, the Erdős-Rényi model (ER) and the Barabasi-Albert model (BA), with parameters chosen to mimic the properties of the real network's member proteins (see Materials and Methods). We found that neither model (each 500 networks) adequately explains the data, yielding networks that have either much higher (ER) or lower (BA) reachability on average (Figure S5A–C). These experiments therefore leave open the statistical nature of the process that generates the network, which we stress currently involves both biological components and experimenter biases, the latter being due to the incomplete nature of the underlying biochemical analyses (many potential edges have not been tested). We next generated two types of edge-shuffled networks, one maintaining in- and out-degree of each node (“Shuffled”) and a second preserving overall in- and out-degree distributions of the network, but not for each node (“Shuffled2”). The mean reachability was lower in the real network (72.09) than in 353 Shuffled networks (70.6% of all 500; average reachability was 73.96 across all 500 networks; see Figure S5D) but higher than all 500 Shuffled2 networks (average 34.8; Figure S5C). Taken together, these results indicate that high reachability emerges quite readily in a network composed of proteins with the measured in- and out-degrees found in a real biological network, such as the protease web described here. In fact, a network without such high reachability—as it is often assumed in biochemistry and cell biology—would be surprising from these results. Importantly, this further suggests that the current biochemical description of cascades and individual proteases working in isolation is unlikely.

### High Connectivity in the Protease Web Is Robust to Possible Annotation Errors

**Figure 5:**
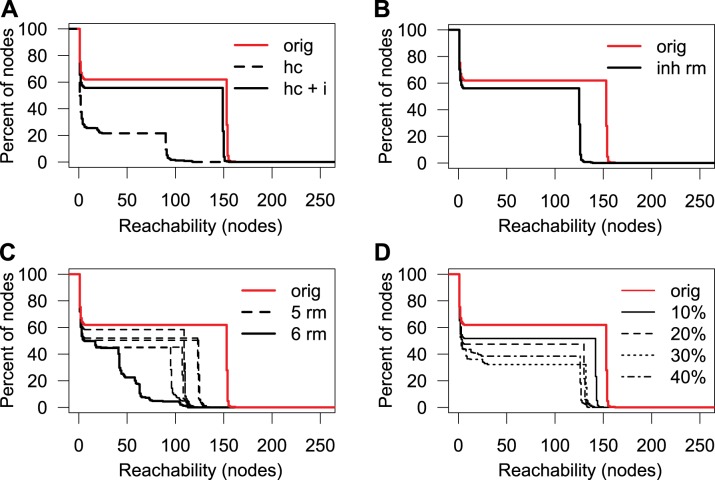
Reachability in the human protease web after various perturbations. Reachability of the largest connected component of the protease web (shown in Figures 2C and 3) after various perturbations. Reachability is plotted as the inverse cumulative function of the percentage of nodes that can reach a given minimum number of nodes in the corresponding network. (A) Reachability in the high confidence network comprised of nodes annotated as having physiological relevance. The reachability distribution of the original network (“orig,” red solid line as also shown in Figure 2C) is compared to networks where edges were removed to create a high confidence network (“hc,” black dashed line) and the high confidence network plus inhibitors (“hc+i,” black solid line). (B) Reachability before (“orig,” red line) and after (“inh rm,” black line) removing edges, reflecting cleavages of inhibitors. Cleavage edges were removed if (i) the inhibitor is annotated to be a serine protease inhibitor and the protease is a serine or cysteine protease or (ii) the inhibitor is A2M or PZP. (C) Reachability after removal of six nodes from the original network (PLG, alpha-1-antitrypsin, A2M, CTSL1, alpha-1-antichymotrypsin, and KLK4). The reachability after removing these six nodes (“6 rm,” black solid line) is compared to the reachability distribution of the original network (“orig,” red line) and to six networks representing each possible combination of keeping one of the six nodes and removing the other five (“5 rm,” black dotted lines), each showing much smaller reduction in reachability. (D) Reachability after removal of random edges. The reachability in the original network (“orig,” red line) compared to networks where 10%, 20%, 30%, or 40% of edges were removed at random. In each case, random edge deletion was carried out 200 times and the worst AUC value was selected for plotting.

To assess reliability of high connectivity in the protease web, which we observed assuming that all cleavage and inhibition data are trustworthy, we addressed the possibility of erroneous data passing through database annotations into our network. A possibility of validating our findings is to compare the network to another second network derived from an orthologous data source. However, MEROPS being the only database of similar coverage, we instead tested whether the same connectivity can be observed by removing nodes in anticipation that some interactions are wrongly annotated. Protease specificity is mostly influenced by three factors: substrate sequence, substrate folding, and the encounter of protease and substrate [46]. In MEROPS/TopFIND, annotations are mostly derived from *in vitro* experiments where a protease is incubated with a substrate. Although some proteases are specific for given substrate sequences, others will cleave a wider range of sequences, but in both cases, possible cleavage sites can be masked in the protein structure of the substrate. Hence, experimental parameters of protease cleavage assays are designed to preserve protein folding and activity of both the protease and substrate in order to prevent unspecific cleavage of denatured substrates. Colocalization of proteases and substrates *in vivo* is an important factor but not unambiguously determinable, with unexpected localization recently revealed [20],[37],[47]–[49],[81]. In addition, most experiments are only performed if it can be assumed that the protease and substrate will colocalize *in vivo*. Assuming that most annotations are correct but individual assignments can be wrong, we randomly and selectively removed edges from the protease web (focusing on the regulatory core, the largest connected component with 255 nodes) to test how reachability is maintained or influenced by such modifications. We utilized the term “physiological relevance,” as annotated in MEROPS and TopFIND, to first create a high-confidence network (abbreviated as “hc” in Figure 5A) by removing all edges that were annotated with physiological relevance other than “yes.” As a consequence, the reachability of the resulting network was markedly decreased (Figure 5A), with the area under the curve (AUC) reduced to 22% of the original network. This was mostly due to the removal of all inhibitors (abbreviated as “i” below) as all 131 human inhibitions in TopFIND have a physiological relevance annotation of “unknown”; that is, their physiological relevance is not annotated in MEROPS from which TopFIND data are largely derived. Upon adding back the inhibitors to the high confidence network (“hc+i”), but still removing all “low confidence” nonphysiological cleavages, high reachability was largely recovered as indicated by an AUC of 88% of the original network. The observation that limiting the cleavages to high-confidence cleavages only barely reduces network connectivity strengthens the result that the protease web is not due to incorrect annotations. Moreover, removing inhibitions from the network severely impacted reachability and thus connectivity, highlighting the essential role of inhibitors in connecting the protease web.

Given the observed importance of inhibitors, we assessed the possibility of incorrect annotation of cleavages of inhibitors. The molecular mechanism of cysteine or serine protease inhibition by serpins involves cleavage of the serpin at its flexible reactive loop, which displays “bait” amino acids. Following cleavage, an induced conformational change leads to entrapment and inactivation of the protease [50],[51]. Because the trap occurs after formation of the acyl intermediate during catalysis, the inhibited serine proteases, but also some cysteine proteases, remain covalently bound to the inhibitor. In contrast, metalloproteinase and aspartic protease cleavage of serpins in the reactive loop does not result in their inhibition, as the nucleophile of these proteases classes is a water molecule. Thus, these proteases are not trapped and therefore escape inhibition, but the serpin is now inactivated. Mechanisms of trapping upon cleavage have also been observed for some metalloprotease inhibitors [52] and for A2M or pregnancy zone protein (PZP, UniProt: P20742), which use a physical trapping mechanism to inhibit all classes of proteases, except exopeptidases [53],[54]. Therefore, annotated cleavages of a protease inhibitor comprise cleavages that reflect either a regulatory inhibition of the protease or a regulatory inactivation cleavage of the inhibitor. To date, this distinction is not annotated in the databases, but is one that we suggest implementing. As a conservative estimate, we removed all cleavages of serpins by serine or cysteine proteases and from any protease to A2M or PZP (“inh rm” in Figure 5B). Therefore 144 edges were deleted from the original 1,238 edges of the largest connected component of the protease web (“orig” in Figure 5B). Notably, this removal only moderately reduced reachability (AUC 74% of original) and preserved a bimodal distribution. Thus, the high connectivity is not a result of unspecific inhibitors. Hence, the observed connectivity in the network is not an artifact attributable to ambiguous annotation of inhibitor cleavage and so further supports the importance of inhibitors in connecting the protease web.

We next assessed the dependence of reachability on individual nodes of the network. By removing each node individually, we found that reachability in the protease web is not dependent on any one single node (Figure S6). Indeed, by iteratively removing all nodes with the highest betweenness from the network, we identified the six most important nodes: plasminogen (PLG; UniProt: P00747), alpha-1-antitrypsin, A2M, cathepsin L1 (CTSL1; UniProt: P07711), alpha-1-antichymotrypsin (also known as serpin A3) (SERPINA3; UniProt: P01011), and kallikrein-4 (KLK4; UniProt: Q9Y5K2) (Figure 5C). Removing all six nodes simultaneously removes 227 edges whereupon this significantly breaks down the bimodal distribution of reachability values, an effect not observed when removing any combination of five out of the six connectors. Thus, high connectivity in the protease web is robust in that it depends not on a single protein, but rather on six important connectors. Furthermore, even after removal of those six nodes the reachability for many proteins remains high with many long paths in the network. Notably, none of these six important nodes are digestive tract proteases, such as trypsin or chymotrypsin, which are broad-acting proteases and ones that might have been expected to form many connections. However, we predict that the identity and number of these key connector proteins will change as more information on the protease web is uploaded to the databases with further experimentation.

Finally, we addressed the possibility of incorrect annotations by removing a fixed percentage of edges, thereby simulating a situation where these edges are incorrect cleavage or inhibition annotations and therefore would have to be removed from the network (Figure 5D). We randomly removed 10%, 20%, 30%, and 40% of all edges (cleavages and inhibitions) 200 times and then plotted the worst case for each experiment. The AUC was reduced to 78%, 65%, 47%, and 52%, respectively, but nonetheless even removal of 40% of edges still preserved the bimodality of the reachability values. Therefore, again the protease web shows a strong resistance to removal of elements, which further increases confidence in the description of a highly connected protease web with inherent robustness to change. This also leads to biological resilience and shows the importance of proteases that can nonetheless be resiliently maintained in genetic deficiencies or pathological perturbations of the system.

### Human Tissue-Specific Protease Webs

**Figure 6:**
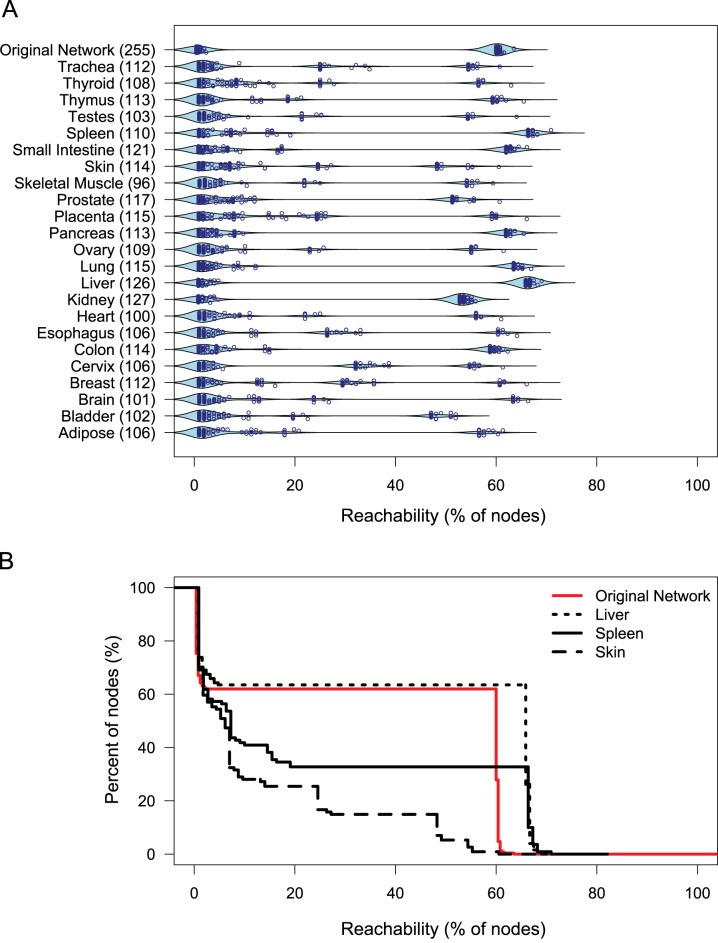
Reachability in tissue-specific protease webs. (A) Beanplot of reachability distributions in the largest connected components of 23 human tissue-specific networks based on gene expression in the corresponding tissues and the original protease web reachability distributions. Overlaid is a scatterplot of the precise values of each node. Numbers in parentheses refer to the size of the network. (B) Inverse cumulative distribution plot of reachability values for skin (dashed black line), spleen (black solid line), liver (dotted black line), and original network (red solid line). Reachability is plotted as an inverse cumulative function of the percentage of nodes that can reach a given minimum percentage of nodes in the corresponding network.

Our analyses suggested that the protease web represents a robust regulatory system of high complexity and flexibility enabling complex patterns of regulation of proteins at the posttranslational level. We next assessed how this system is implemented *in vivo* where only a fraction of proteases and inhibitors is expressed or active at the same time in the same cell, compartment, or tissue. We constructed tissue-specific networks based on protease and inhibitor gene expression levels in 23 different human tissues quantified by CLIP-CHIP microarray (Kappelhoff et al., unpublished data available at http://clipserve.clip.ubc.ca/supplements/protease-web). We used negative control spots on this microarray to define a threshold of expression at detectable levels and then limited networks to those proteins expressed above this threshold. We next plotted the reachability of the nodes in the largest connected component of the resulting networks for all 23 tissue-specific protease webs (Figure 6A). Figure 6B shows liver, spleen, and skin results in more detail. Although most tissue-specific networks (e.g., skin) show low reachability values, some preserve the strong connectivity of the original network totally (e.g., kidney and liver) or partially (e.g., spleen, small intestine, pancreas, lung, colon). Notably, the tissue-specific networks also show that reachability is highly dependent on expression of the same six network connectors shown in Figure 5C (Figure S7).

### Evidence for the Protease Web in Other Data

**Figure 7:**
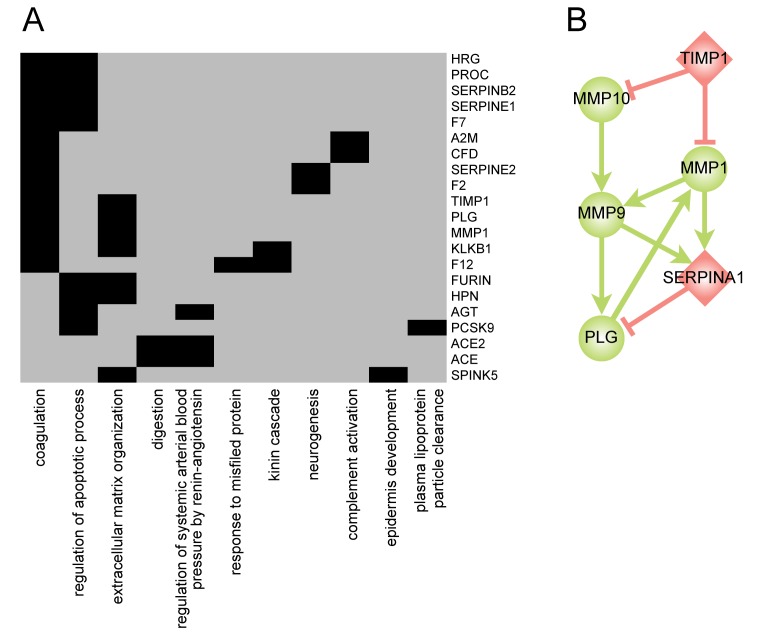
Proteases and their inhibitors involved in multiple, discreet biological processes. (A) A matrix showing the annotation of proteases and inhibitors with selected, protease-specific biological processes based on Gene Ontology [61]. Proteins annotated with more than one term are displayed. (B) A subnetwork of the protease web connecting TIMP1 to coagulation: TIMP1 (UniProt: P01033) inhibits MMP10 (UniProt: P09238) and MMP1 (UniProt: P03956), which both cleave and activate MMP9 (UniProt: P14780), which cleaves PLG (UniProt: P00747). Similarly, MMP1 and MMP9 cleave and inactivate serpin A1 (UniProt: P01009), which is an inhibitor of PLG.

In agreement with our findings based on biochemical interactions, general biological literature also shows that proteases and their inhibitors can be involved in multiple biological processes (Figure 7A). It is easy to imagine that this multifunctionality is partly due to the interplay in the protease web. Indeed most of the proteins in Figure 7A are found in the strongly connected component of our protease web, indicating that they serve in connecting different biological processes. One example is TIMP1 (UniProt: P01033). Protein expression levels of TIMP1, an MMP inhibitor mainly involved in extracellular matrix remodeling and organization, were found associated with hemostasis [55]. This finding, which is derived from orthogonal data to the protease web, primed us to search for connections linking TIMP1 to coagulation factors, which we could indeed identify (Figure 7B). Together, these provide a plausible mechanism of action of TIMP1 and hence MMPs on coagulation and could explain the association observed. Hence, the protease web can be used to explain multifunctionality of proteases, which in turn strengthens our conclusion of a large interplay between proteases.

### Using the Protease Web to Decipher *in Vivo* Network Effects

We were able to test the utility of our graph representation of the protease web by deciphering a previously inexplicable result *in vivo*. We analyzed the MMP8-dependent cleavage of the murine chemokine C-X-C motif chemokine 5 (CXCL5, UniProt: P50228), also known as lipopolysaccharide (LPS)-induced C-X-C chemokine LIX (LIX). LIX is a potent chemoattractant chemokine for polymorphonuclear (PMN) leukocytes, and MMP8 (UniProt: O70138) is PMN specific. It was previously demonstrated in an *in vivo* airpouch model that MMP8 knockout mice showed reduced PMN migration in response to LPS [56]. This was attributed to MMP8 processing and activation of LIX at position Ser^4^↓Val^5^, with a second cleavage at Lys^79^↓Arg^80^ of the 92-residue protein. Indeed the MMP8-truncated activated form of LIX (5–79) showed equal cell migration in wild-type and knockout mice, validating LIX as a physiological MMP8-dependent mechanism for promoting neutrophil infiltration *in vivo*. However, a neoepitope antibody specific to the MMP8-generated neo-N terminus failed to detect truncations at Ser^4^↓Val^5^ in the airpouch model. Thus, cleavage of LIX is a MMP8-*dependent* but MMP8-*indirect* event *in vivo* that could not be explained, prompting a further analysis of alternate MMP8-dependent proteolytic pathways predicted using our representation of the protease web.

**Figure 8:**
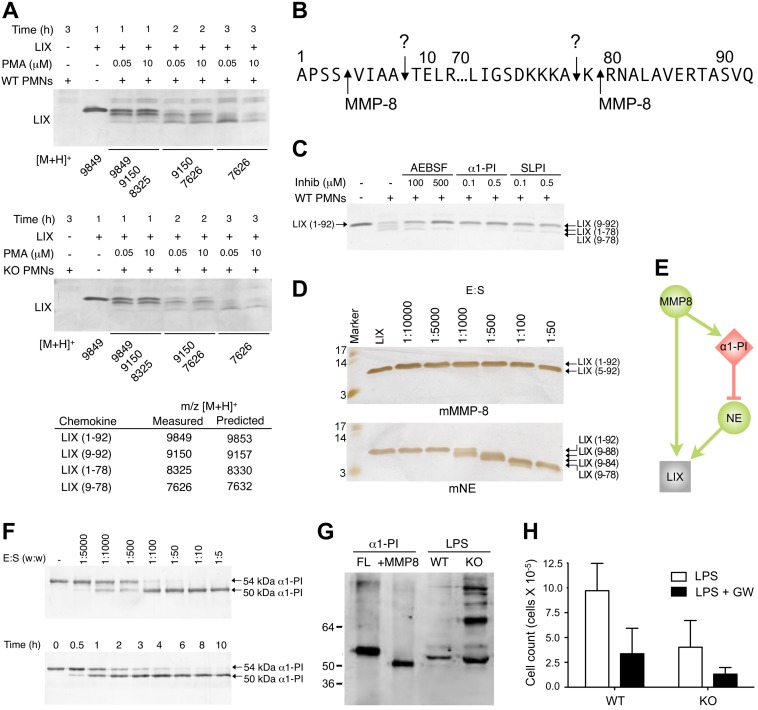
Protease web affects validation *in vivo*. (A) Tris-Tricine 15% SDS-PAGE and MALDI-TOF mass spectrometry analyses of LIX cleavage following incubation with wild-type (WT) or MMP8-deficient (KO) murine polymorphonuclear leukocytes (PMNs) for up to 3 h after PMA stimulation to release PMN proteases to the culture medium. (B) Sequence of the N- and C-terminal regions of LIX with cleavage sites by PMN MMP8 and the unknown protease (?) annotated. (C) Tris-Tricine SDS-PAGE analysis of LIX cleavage by PMNs after addition of protease inhibitors: AEBSF, 2-aminoethyl benzenesulfonyl fluoride hydrochloride; α1-PI, α1-proteinase inhibitor; SLPI, secreted leukocyte proteinase inhibitor. (D) LIX cleavage by murine (m) MMP8 and murine neutrophil elastase (mNE) analyzed by 15% Tris-Tricine SDS-PAGE analysis and MALDI-ToF mass spectrometry. E:S, enzyme-to-substrate ratio; “Marker,” molecular weight markers as indicated. (E) Network effects on LIX cleavage. Proteases are green, inhibitors red, and other substrate proteins are grey. Edges are cleavages (green, with arrow head) or inhibitions (red, with “T” head). (F) MMP8 cleavage of α1-proteinase inhibitor (α1-PI). The serine protease inhibitor α1-PI was incubated with MMP8 for 16 h at 37°C in 50 mM Tris, 200 mM NaCl, 5 mM CaCl_2_, pH 7.4 containing 1 mM APMA. The enzyme-to-substrate (E:S) ratio ranged from 1∶5 to 1∶5,000 (w:w). Reactions were visualized on a 10% SDS-PAGE (silver stained). Below, a time course of MMP8 cleavage of α1-PI at 1∶50 (w:w) E:S ratio. (G) Bronchioalveolar lavage of mice stimulated with LPS. WT, wild-type mouse; KO, MMP8 knockout mouse. LPS (2 µg) was instilled in the lungs of female mice, and after 48 h, the mice were sacrificed and the lungs lavaged with PBS. Cell-free bronchioalveolar lavage from three mice was pooled and concentrated by acetone precipitation. α1-PI detection was with Alexa-conjugated antibodies (Molecular Probes) on the LiCOR Odyssey. (H) Numbers of PMNs in the bronchioalveolar lavage after LPS stimulation with (*n* = 3) and without (*n* = 3) instillation of GW311616 (GW), a specific neutrophil elastase inhibitor.

To examine the importance of neutrophil-derived MMP8 in LIX processing and activation, we isolated bone marrow neutrophils from wild-type and MMP8 knockout mice. Neutrophils were stimulated with phorbol myristate acetate (PMA) followed by incubation of the activated neutrophils with chemokine for up to 3 h. Truncations of LIX generating the bioactive products LIX (9–92) and LIX (9–78), as determined by MALDI-TOF mass spectrometry from the still inactive form LIX (1–78), were readily apparent, even after only 1 h of incubation (Figure 8A). However, both the MMP8 knockout and wild-type neutrophils showed identical cleavage sites (Ala^8^↓Thr^9^ and Ala^78^↓Lys^79^) and cleavage kinetics. Because these sites differ from the MMP8 cleavage sites (Figures S8, S9, and 8B), MMP8 is not the dominant neutrophil protease cleaving LIX in the cellular context. Investigating protease web effects that may account for this, we found that LIX cleavage by neutrophils was inhibited by the serine protease inhibitor 2-aminoethyl benzenesulfonyl fluoride hydrochloride (Figure 8C). This showed that one or more of the four serine proteases in neutrophils—neutrophil elastase (UniProt: Q3UP87), cathepsin G (UniProt: P28293) [57], proteinase-3 (UniProt: Q61096), or the recently described neutrophil serine proteinase 4 (UniProt: Q14B24) [58]—were responsible for LIX cleavage. Using low concentrations of the endogenous serine proteinase inhibitors α1-proteinase inhibitor (α1-PI, UniProt: P07758) [21] and secreted leukocyte proteinase inhibitor (SLPI, UniProt: P97430) (Figure 8C), we excluded proteinase-3 and neutral serine proteinase 4 as candidates, as SLPI does not inhibit these proteinases [58],[59]. Moreover, neutral serine proteinase 4 has a stringent substrate specificity that does not fit our observed cleavage sites. Cathepsin G did not cut after Ala^8^ and required high enzyme concentrations (>100 nM) in generating the C-terminal cleavage (Figure S9) as it was inefficient with a k_cat_/K_M_ 60 M^−1^ s^−1^. Thus, neutrophil elastase was the strongest candidate, and indeed 1 nM elastase efficiently cleaved LIX with a k_cat_/K_M_ 1,200 M^−1^ s^−1^ at Ala^8^↓Thr^9^ and Ala^78^↓Lys^79^ (Figures 8D, S8, and S9). Because MMP8 cleaves N-terminal to the Ala^8^↓Thr^9^ elastase site and C-terminal to the Ala^78^↓Lys^79^ elastase site, truncations by elastase will remove evidence of any MMP8 cleavage. Furthermore, MMP8 is less efficient (k_cat_/K_M_ 600 M^−1^ s^−1^) than elastase in cleaving LIX. Thus, elastase is the dominant protease for LIX cleavage by neutrophils *in vivo*.

To explain the paradoxical result that in the *Mmp8* ^−/−^ mouse LIX is not cleaved *in vivo* despite the presence of neutrophil elastase, we employed path finding in the protease web to identify potential regulatory effects from MMP8 on neutrophil elastase. Although no path was found in the murine network, the more extensive human network contains a path that had potential to explain this perplexing result (Figure 8E). Human MMP8 is known to cleave and inactivate human α1-PI [21], the potent inhibitor of neutrophil elastase, but SLPI is resistant to MMP8 cleavage [60]. We verified α1-PI cleavage by MMP8 using mouse proteins for the first time at various enzyme-to-substrate ratios and in time course experiments (Figure 8F) from which we found that murine MMP8 efficiently cleaves and inactivates murine α1-PI *in vitro* with a k_cat_/K_M_ 7.7×10^3^ M^−1^ s^−1^.

We next validated the *in vitro* results *in vivo*. In murine bronchioalveolar lavage collected following 24 h of treatment with LPS, both the full-length and high molecular weight forms of α1-PI, which were present as inhibitor-serine protease complexes, were greatly enhanced in *Mmp8* ^−/−^ mice compared to wild type (Figure 8G). Together, these *in vitro* and *in vivo* data show that efficient cleavage of α1-PI occurs by MMP8 *in vivo* and indicates the importance of MMP8 in modulating the balance of functional α1-PI protein and activity *in vivo* and hence elastase activity. This result further shows that MMP9, which also cleaves alpha1-PI in vitro, does not functionally compensate for MMP8 in vivo. This is despite MMP9 being in the same cytosolic granules as MMP8 and being present at elevated concentrations in the neutrophils from the MMP8 knock out mouse. Finally, we confirmed neutrophil elastase-dependent LIX cleavage *in vivo* using a specific neutrophil elastase chemical inhibitor (GW311616). Specific elastase inhibition reduced the relative numbers of neutrophils in wild-type mouse bronchioalveolar lavage similar to the decrease in cell migration in the MMP8 knockout versus the wild-type mouse bronchioalveolar lavage (Figure 8H). We conclude that MMP8 cleaves and inactivates α1-PI *in vivo* acting as the “metallo-serpin” switch leading to increased neutrophil elastase activity and LIX activation, which thereby promotes neutrophil infiltration *in vivo*. Evidence of LIX cleavage by MMP8 is lost following elastase cleavage *in vivo*, which is also catalytically more efficient than MMP8. Thus, the protease web enabled deconvolution of a complex biologically relevant proteolytic event and in turn formulation of a testable hypothesis that was confirmed *in vitro* and *in vivo*.

## Discussion

To our knowledge, this is the first systematic bioinformatics analysis of the extent and structure of the protease web. We assembled *in silico* networks comprising all biochemically annotated interactions between proteases and their inhibitors, which therefore represent the potential of regulation among proteases based on current biochemical data. By representing the human protease web as a graph, we show the depth of how proteases and inhibitors regulate each other across families and even catalytic classes. Thus, known cascades and proteases do not act in isolation, as often assumed, but crosstalk extensively. The structure of the human protease web is not cascade-like and hierarchical but multidirectional with connections between top and bottom proteins of known cascades with six proteases and inhibitors identified as key connectors in this network. Although other connectors might be identified in future versions of the network, this shows how regulatory switches, especially inhibitors, tether subnetworks of the overall network. Notably, the observed potential for regulatory crosstalk between proteases and inhibitors is not an artifact of data annotation as it persists robustly despite various perturbations we tested (Figure 5). On the contrary, the extent of such crosstalk is an underestimation because current data on protease cleavage and inhibition are largely incomplete.

As high-throughput terminomics analyses continue to massively add new information, more connections will undoubtedly be found, thereby further increasing the observed connectivity. In fact, a decrease in connectivity can only occur if current annotations are proven wrong and are corrected by removing edges from the network. However, we demonstrated that connectivity in the protease web is highly robust against such modifications, further validating the existence of a pervasive network of proteases and inhibitors embedded in different proteomes. Investigating tissue-specific implementations of the protease web, we found that gene expression shapes the protease web specifically in various tissues. Thus, subnetworks of the entire network are active at any place and time in different tissues. Some human tissues exhibit a protease web with connectivity close to the global network, further validating the existence of such a network *in vivo*. Mouse annotations are currently focused on few proteases and can therefore not yet display large-scale network features. Despite this and the current lower connectivity in the murine network (Figure 2C), we expect that with further annotations the murine network will morph to form more of a multidirectional, highly connected structure similar to the described human network.

The utility of the protease network as a concept and as a tool was demonstrated in successfully deciphering a paradoxical *in vivo* result involving cleavage of the murine chemokine LIX by neutrophils, an important inflammatory cell in innate immunity, which had been previously shown to be a substrate of the neutrophil-specific MMP8 [56]. Our analyses showed that even though MMP8 cleaves LIX *in vitro* and in the *Mmp8* ^−/−^ mouse LIX cleavage is also reduced, it was not cut by MMP8 *in vivo*. Rather, we identified neutrophil elastase as the relevant protease *in vivo*. Path finding in the protease web enabled us to then prove that MMP8 potently but indirectly facilitated LIX cleavage through direct MMP8 cleavage and inactivation of the elastase inhibitor α1-PI in cellular contexts and *in vivo*. Thus, combining individual interactions stored in TopFIND/MEROPS through interrogation of the protease web by random and directed walks generated a testable hypothesis that was experimentally validated. This revealed the mechanistic importance of MMP8 in mediating the cleavage of LIX—not directly as observed *in vitro*, but indirectly by enabling elastase activity through removal of the biologically relevant blocking inhibitor, thus forming a metallo-serpin switch to regulate the concentrations of active *versus* inactive α1-PI *in vivo*. The biological outcome of path walking in the network will depend on the relative concentrations of the individual nodes in different tissues or tissue conditions and pathologies. Thus, what is biological meaningful in one situation may not be in another and so requires experimental validation, as we performed here. Hence, the overall workflow of path prediction and validation can now be transferred to other investigations of complex *in vivo* protease biology.

### Principles of Regulation in the Protease Web

Critical control of protease activity is exerted at the protein level. Proteases from one class (e.g., metalloproteases) frequently cleave proteases from other classes (e.g., serine proteases) or their cognate inhibitors (serpins), and subnetworks can thereby be activated or inactivated. In this process, we found that protease inhibitors take an important connecting role in the web—they are highly enriched as substrates of all classes of proteases and removal of inhibition strongly decreases reachability of all nodes in the network. Protease inhibitors often lack specificity and inhibit families of proteases rather than just individual enzymes. Thus, inhibitors function as key on/off switches of entire subnetworks within the protease web, enabling rapid and efficient activation of proteolytic processes upon their cleavage. We provided a new example of a metallo-serpin switch controlling chemokine activation. As an important biological consequence of this, removal of inhibition is therefore recognized to be as important as zymogen activation in cascades in controlling proteolysis. Indeed this was recently demonstrated in skin inflammation *in vivo*, where MMP2 was found to cleave and inactivate serpin G1, also known as complement C1 inhibitor [13]. Dynamically regulating the activity levels of serpin G1 inhibition allowed complement activation to cascade, which otherwise was greatly reduced in the *Mmp2* ^−/−^ mouse, where excess amounts of intact functional serpin G1 were proteomically quantified by TAILS terminomics. The central role of this metallo-serpin inhibitor switch in the protease web was further shown in the regulation of another subnetwork involving plasma kallikrein cleavage of kininogen to release the vasoactive peptide bradykinin. The network representation of the protease web emphasizes that proteases of one family and class can markedly regulate the activity of proteases from different families and classes.

### Applicability of the Protease Web

Understanding a complex biological network, such as the protease web, can only be achieved via systematic storing and sharing of biochemical information in order to enable network-based predictions to generate testable hypotheses. Applying this strategy, we gained *in silico* insights into *in vivo* processes and validated these biochemically, in culture and *in vivo*. We forecast that through further identification and biochemical characterization of cleavage and inhibition events, the representation of protease interactions can be improved to strengthen its predictive power. The resulting network could then be used to simulate the effects of protease and inhibitor knockouts and protease drug targeting in disease, which will enhance confidence of targeting the correct protease and thereby increase the success rate of clinical trials by reducing unexpected side effects.

In conclusion, our analysis of the protease web reveals a multidirectional rather than a hierarchical structure, as has been proposed [40], with deep connections in regulation of the proteome by specific proteolytic processing in addition to degradation. As the structure of the human protease web is multidirectional rather than cascade-like and hierarchical, it has high connectivity that is robust to change. Biologically this implies that regulation by proteolysis is a consistent and pervasive force in all tissues. In comparison to phosphorylation, which is limited to intracellular proteins and pathways, proteolysis affects all proteins and pathways inside and outside the cell, and it is irreversible and pervasive and needs to be considered in functional analyses of the proteome.

## Materials and Methods

### Protease Web Data

Tables containing proteases and their substrates (cleavages) and protease inhibitors and their target proteases (inhibitions) as well as tables mapping UniProt IDs to MEROPS IDs and gene names were collected from the TopFIND MySQL database (http://clipserve.clip.ubc.ca/topfind/; downloaded January 15, 2012).

### Classifying Proteases and Inhibitors

Proteases were classified based on their MEROPS IDs in TopFIND. Determining the inhibitor class specificity of human protease inhibitors was performed by downloading lists of UniProt ACs for Gene Ontology [61] annotations cysteine-type (GO:0004869, *n* = 49 proteins), metallo- (GO:0008191, *n* = 11 proteins), or serine-type (GO:0004867, *n* = 95 proteins) endopeptidase inhibitor from neXtProt [62] on May 24, 2012. A term “aspartic-type endopeptidase inhibitor” (GO:0019828) exists, but no proteins are annotated with this term. Inhibitors were labeled “broad” if they are annotated to inhibit more than one class of protease based on (i) their GO terms from neXtProt or (ii) their annotated inhibitions from TopFIND.

### Network Construction and Analysis

The network representation of cleavages and inhibitions was obtained via R [63] scripts, heavily relying on the use of the *igraph* library [64]. Proteins are represented as nodes. Cleavages are represented as directed edges from the proteases node to the substrate node. Accordingly, inhibitions were represented as directed edges from the inhibitor to the inhibited protease. Reachability of a node was calculated by counting all proteins where a shortest path can be found using the *shortest.path* function of *igraph*. Betweenness of nodes was calculated using the *betweenness* function of the *igraph* package. By recalculating betweenness after removing each node, the iterative identification of nodes with the highest betweenness was performed. Paths from MMP8 to neutrophil elastase were identified in the network using the *get.all.shortest.paths* function of the *igraph* package. Erdős-Rényi networks with the same number of nodes and edges as the original graph were generated using the *erdos.renyi.game* function of the *igraph* package, and Barabasi-Albert networks were generated with the *barabasi.game* function, forcing the same out-degree distribution as the protease web. Edge-shuffled random graphs were generated using the *degree.sequence.game* function once keeping out- and in-degree distributions the same so that each node has the same in- and out-degree as in the original network (Shuffled) and once shuffling those distributions before passing them to the method (Shuffled2). Inverse empirical cumulative distribution functions were calculated and plotted using an inverted version of the empirical cumulative function “e*cdf*” in R. The AUC was calculated by calling the *integrate* function in R on the cumulative function.

### Mapping Mouse to Human Proteins

Mouse and human networks were compared by identifying connections, which occur between homologous proteins. The homology mapping between UniProt ACs of the two species was performed by mapping UniProt ACs to Ensembl protein IDs via the Ensembl database of the *biomaRt* package [65] in R obtained from Bioconductor [66]. The homology mapping between Ensembl protein IDs was performed using the InParanoid [67] database via the *hom.Hs.inp.db* [68] package in R/Bioconductor.

### Network Figures

Network figures were plotted using Cytoscape 2.8.3 [69].

### Involvement of Proteases and Inhibitors in Biological Processes

Proteins involved in selected, protease-specific biological processes were identified by obtaining Gene Ontology [61] annotation of proteins using the *org.Hs.eg.db* package [70] in R/Bioconductor on August 8, 2013.

### In Vivo N-Terminomics Data of Murine Skin

N-terminal cleavage sites in normal and inflamed murine skin were obtained from Supplementary table S8 from [13].

### Analysis of Protease and Inhibitor Expression in 23 Human Tissues

The data for the analysis of the protease and inhibitor expression profile was achieved by analysis of commercially available RNAs from 23 different healthy human tissues on the protease- and inhibitor-specific oligonucleotide-based CLIP-CHIP microarray [71]. Data from 84 CLIP-CHIP microarrays representing biological and technical replicates of antisense RNA of these tissues were used, and average signal intensity values (A-Value) of each gene were combined. An expression cutoff was determined at an A-Value of 7.5, where 95% of the intensities of the negative oligonucleotide probes on the microarray were below this cutoff (data are available at http://clipserve.clip.ubc.ca/supplements/protease-web).

#### Chemokines, proteinases, and inhibitors

All chemokines were synthesized using tBoc (tertiary butyloxycarbonyl) solid phase chemistry as described previously [72]. Recombinant human and murine MMP8 were expressed and purified as described previously [73]. Human neutrophil elastase and cathepsin G were purchased from Elastin Products Company and Calbiochem, respectively. Murine neutrophil elastase was kindly provided by Dr. Dieter Jenne (Max Plank Institute of Neurobiology, Martinsried). The 2-aminoethyl benzenesulfonyl fluoride hydrochloride and α1-proteinase inhibitor were from Sigma, and SLPI was from ICN Biomedicals. The synthetic neutrophil elastase inhibitor GW311616 was from Tocris Bioscience.

#### Animals

Mice deficient in MMP8 on a C57BL6/J×129 S background were provided by Dr. S. Shapiro (Boston, MA). Animal breeding and experimental procedures were approved by the Animal Care Committee of the University of British Columbia. Mice 6 to 8 wk old, segregated according to sex, were used for all experiments.

#### Neutrophil isolation and LIX cleavage assays

Murine neutrophils were isolated from bone marrow by flushing of fibulas and tibias. Neutrophils were separated on a density gradient comprised of Histopaque 1077 layered on top of Histopaque 1119 according to the manufacturer's instructions (Sigma) followed by washing with Hanks Balanced Salt Solution. Neutrophil purity and viability were consistently determined to be >90%. Neutrophils were activated with 50 nM phorbol 12-myristate 13-acetate (Sigma), unless indicated otherwise. Neutrophils (1×10^6^ cells) were incubated with 10 mg LIX for up to 4 h in Dulbecco's Modified Eagle Medium at 37°C. Inhibitors were preincubated with cells for 30 min at 37°C prior to the addition of chemokine. Cells were removed by centrifugation (500×*g*, 5 min) at the desired time points, and supernatants were analyzed as described below by MALDI-TOF mass spectrometry and Tris-Tricine SDS-PAGE.

#### LIX cleavage assays

Analysis of substrate cleavage by isolated proteases was performed at enzyme/chemokine (E:S) ratios from 1∶10,000 up to 1∶50 (mol:mol) for 16 h at 37°C in assay buffer (50 mM Tris, 200 mM NaCl, 5 mM CaCl_2_, pH 7.4). MMP8 was activated by 1 mM 4-aminophenylmercuric acetate (Sigma). Digests were spotted on MALDI target plates with sinapinic acid for MALDI-TOF analysis or terminated by adding SDS-PAGE sample buffer. Reaction products were analyzed by 15% Tris-Tricine SDS-PAGE and silver stained. Specificity constants (k_cat_/K_M_) of cleavage were determined by densitometry as described previously [74]. The mass-to-charge ratios (m/z) with +1 ionization ([M+H]^+^) were determined on a Voyager-DE STR Biospectrometry Workstation (ABI). Mass spectrometry data were deconvoluted to identify the substrate cleavage sites. Molecular weight prediction was obtained using the “*Compute pI/Mw tool*” [75] on expasy.org.

## Supporting Information

Figure S1 Protease networks in mouse and human. Networks of all proteases (green circles), protease inhibitors (red diamonds), and protease substrates (grey squares), which take part in any cleavage or inhibition reaction annotated in MEROPS/TopFIND. Networks are shown for human (A) and mouse (B). To resolve individual nodes and edges, click to zoom. Proteins are designated by their UniProt gene names.
(EPS)

Figure S2 Annotation biases in protease substrate identification. Out-degree of protease and inhibitor proteins with an out-degree of 1 or greater in the human and mouse data. Out-degree is the sum of cleavages catalyzed by a protease or inhibitions caused by a protease inhibitor. Proteins (nodes) are sorted by their out-degree. Human values are in red; mouse values are in blue.
(EPS)

Figure S3 Human proteases are overrepresented as substrates. Percentage of proteases and inhibitors that are known substrates. The percentages of all UniProt/Swiss-Prot proteins with an annotated MEROPS ID indicating they are proteases or inhibitors are shown as “theoretical.” “TopFIND” refers to the percentage of all substrates that are proteases or inhibitors found in the TopFIND database. The percentage of proteases or inhibitors (proteins with a MEROPS ID) amongst all internal neo-N termini in a recent TAILS analysis of murine skin [13] are referred to as “murine TAILS data.”
(EPS)

Figure S4 New connections in known proteolytic pathways. (A) Coagulation, (B) complement system, (C) apoptosis, and (D) kallikreins are shown with connections as they are in the network. Proteases are represented as green circles and inhibitors as red diamonds. Edges are cleavages (green, with arrow head) and inhibitions (red, with “T” head). Edges of originally defined pathways are solid, and additional edges are dotted. (A) Coagulation factors XII, XI, X, IX, VII, and V that form the clot (UniProt gene names: F12, F11, F10, F9, F7, and F2) are connected as originally described [30]. This figure also shows PLG, tissue-type, and urokinase-type PLG activators involved in fibrinolysis (PLG, PLAU, and PLAT) [34] and many connections between those proteins, which were not classically described. (B) The main complement cascade of proteins C1R, C1S, C2, C3, and C5 of the classical pathway, as well as cofactors from the alternative pathway complement factors D, B, and I (UniProt gene names: CFD, CFB, and CFI) [26]. Additional connections not originally described are with the lectin pathway activators mannose-binding lectin serine protease 1 and 2 (MASP1 and MASP2) [28] and the plasma protease C1 inhibitor (SERPING1) [27]. (C) The network contains connections between initiator caspases 8, 9, and 10 (UniProt gene names: CASP8, CASP9, and CASP10), and their cleavage of effector caspases 3 and 7 (CASP3 and CASP7) and caspase 6 (CASP6) as described in [33]. The network also contains caspases 4 (CASP4) and interactions with apoptosis protease inhibitors (BIRC7, BIRC8, and XIAP). (D) Kallikreins of the semen liquefaction cascade are connected as described previously [31] with the protease network showing many additional connections.
(EPS)

Figure S5 The protease web compared to random networks. (A) Out-degree and (B) in-degree of nodes in the protease web (“Real network”) compared to the Barabasi-Albert (BA) and Erdős-Rényi (ER) model networks (averaged over 500 networks). A small constant (0.001) was added to enable log/log plots. (C) Mean reachability of nodes in 500 networks generated from each BA and ER model, and two different edge-shuffling methods (boxplots) compared to the protease web (red line). (D) Mean reachability in the protease web (red line) compared to the mean reachability of 500 edge-shuffled networks (black density curve).
(EPS)

Figure S6 Reachability in the network does not depend on one single node. (A) High reachability is maintained after removal of single nodes from the network. The original protease web (“orig,” red line) is compared to 255 modified networks, each of which is missing one of the 255 nodes of the original network (“1 rm,” black lines). (B) The AUC for the 255 modified networks (histogram) compared to the AUC under the original network (red vertical line).
(EPS)

Figure S7 Reachability in the protease web strongly depends on the presence of six important nodes. Reachability plotted against the presence of the six important proteins identified in Figure 5C (PLG, SERPINA1, A2M, CTSL1, SERPINA3, and KLK4) for the 23 tissue-specific networks. The AUC of the inverse cumulative function of reachability values in each tissue-specific network (*x*-axis) was plotted against the count of important proteins (out of six) present in each network (on the *y*-axis).
(EPS)

Figure S8 MALDI-TOF analysis of LIX cleavage by MMP8 and neutrophil elastase. LIX cleavage products from Figure 8D analyzed by MALDI-TOF mass spectrometry. Analysis of LIX alone (LIX 1–92) was compared to the LIX cleavage products at E:S ratios of 1∶5,000, 1∶500, and 1∶50 for murine MMP8 on the left and murine neutrophil elastase (NE) on the right. MMP8 and NE are not observed in this m/z range of the spectra.
(EPS)

Figure S9 MMP8, neutrophil elastase, and cathepsin G cleavage of LIX. (A) LIX cleavage by murine (m) and human (h) proteases MMP8, neutrophil elastase (NE), and cathepsin G (CATG) analyzed by 15% Tris-Tricine SDS-PAGE analysis and MALDI-TOF mass spectrometry. Resolution of mMMP8 cleavage products was technically difficult to show by gel electrophoresis and so we relied upon the data generated by MALDI-TOF mass spectrometry (Figure S8). E:S, enzyme to substrate ratio; “Marker,” molecular weight markers as indicated. (B) Sequence of the N- and C-terminal regions of LIX with major protease cleavage sites annotated as determined by MALDI-TOF mass spectrometry. Sites for MMP8 and NE were found for both human and murine enzymes; mNE are unique for the murine neutrophil elastase.
(TIFF)

Table S1 List of nodes with highest reachability and betweenness in the network.
(DOCX)

## Abbreviations

α1-PI: α1-proteinase inhibitor
AUC: area under the curve
LIX: cytokine LIX (mCXCL5)
MALDI: matrix-assisted laser desorption/ionization
MMP: matrix metalloproteinase
PMN: polymorphonuclear leukocyte
SDS-PAGE: sodium dodecyl sulfate polyacrylamide gel electrophoresis
TOF: time of flight
