# Supplementary figures and images for: Network Analyses Reveal Pervasive Functional Regulation Between Proteases in the Human Protease Web
Source: PLoS Biol. 2014 May 27;12(5):e1001869. doi: 10.1371/journal.pbio.1001869 (PMC4035269; doi:10.1371/journal.pbio.1001869)

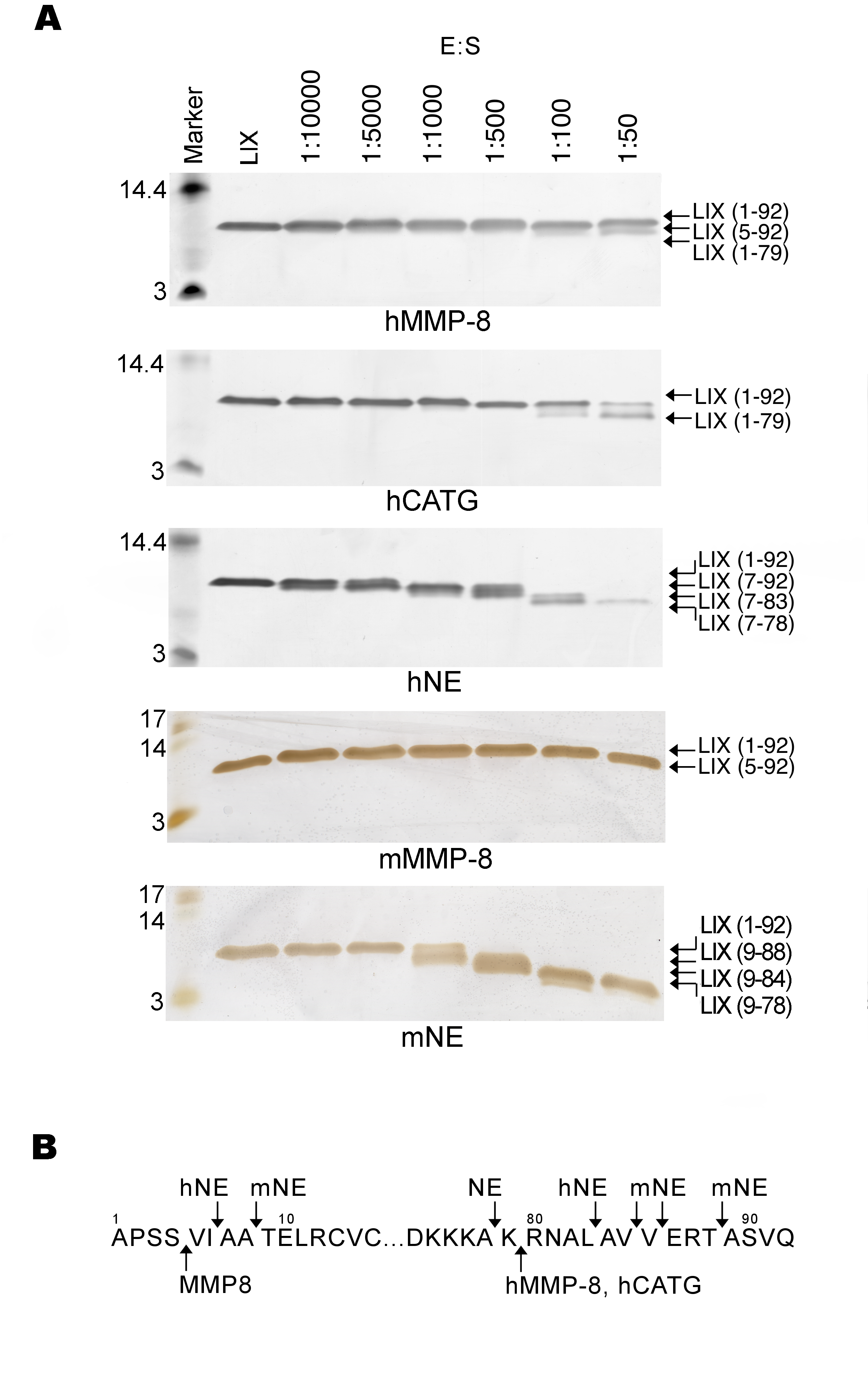

Supplement: Figure S9 — MMP8, neutrophil elastase, and cathepsin G cleavage of LIX. (A) LIX cleavage by murine (m) and human (h) proteases MMP8, neutrophil elastase (NE), and cathepsin G (CATG) analyzed by 15% Tris-Tricine SDS-PAGE analysis and MALDI-TOF mass spectrometry. Resolution of mMMP8 cleavage products was technically difficult to show by gel electrophoresis and so we relied upon the data generated by MALDI-TOF mass spectrometry (Figure S8). E:S, enzyme to substrate ratio; “Marker,” molecular weight markers as indicated. (B) Sequence of the N- and C-terminal regions of LIX with major protease cleavage sites annotated as determined by MALDI-TOF mass spectrometry. Sites for MMP8 and NE were found for both human and murine enzymes; mNE are unique for the murine neutrophil elastase. (TIFF) [file pbio.1001869.s009.tif]
