# Supplementary material for: Network Analyses Reveal Pervasive Functional Regulation Between Proteases in the Human Protease Web
Source: PLoS Biol. 2014 May 27;12(5):e1001869. doi: 10.1371/journal.pbio.1001869 (PMC4035269; doi:10.1371/journal.pbio.1001869)
Supplement: Table S1 — List of nodes with highest reachability and betweenness in the network. (DOCX) [file pbio.1001869.s010.docx]

**Table S1.** List of nodes with highest reachability and betweenness in the network

| - - 1. **Gene name** | - - 1. **MEROPS ID** | - - 1. **Reachability** | - - 1. **Betweenness** | - - 1. **Out-degree** | - - 1. **In-degree** |
| --- | --- | --- | --- | --- | --- |
| - - 1. **FURIN** | - - 1. S08.071 | - - 1. 162 | 0 | 22 | 3 |
| - - 1. **CST6** | - - 1. I25.006 | - - 1. 157 | 0 | 4 | 0 |
| - - 1. **PIGK** | - - 1. C13.005 | - - 1. 156 | 0 | 2 | 0 |
| - - 1. **TMPRSS15** | - - 1. S01.156 | - - 1. 155 | 0 | 2 | 0 |
| - - 1. **HTRA2** | - - 1. S01.278 | - - 1. 155 | 0 | 9 | 4 |
| - - 1. **MMP11** | - - 1. M10.007 | - - 1. 155 | 43 | 7 | 3 |
| - - 1. **PCSK5** | - - 1. S08.076 | - - 1. 155 | 0 | 4 | 0 |
| **PCSK7** | - - 1. S08.077 | - - 1. 155 | 0 | 1 | 0 |
| - - 1. **CTSL3** | - - 1. I29.001 | - - 1. 155 | 0 | 3 | 0 |
| - - 1. **CSTA** | - - 1. I25.001 | - - 1. 155 | 0 | 5 | 0 |
| - - 1. **PLG** | - - 1. S01.233 | - - 1. 153 | 7939 | 40 | 27 |
| - - 1. **A2M** | - - 1. I39.001 | - - 1. 153 | 6980 | 27 | 16 |
| - - 1. **CTSL1** | - - 1. C01.032 | - - 1. 153 | 5215 | 29 | 13 |
| - - 1. **APP** | - - 1. I02.015 | - - 1. 153 | 4920 | 5 | 102 |
| - - 1. **SERPINA1** | - - 1. I04.001 | - - 1. 153 | 4400 | 10 | 25 |
| - - 1. **KNG1** | - - 1. I25.016 | - - 1. 153 | 4183 | 1 | 55 |
| - - 1. **KLK4** | - - 1. S01.251 | - - 1. 153 | 3770 | 32 | 6 |
| **CASP3** | - - 1. C14.003 | - - 1. 153 | 2529 | 24 | 13 |
| - - 1. **ELANE** | - - 1. S01.131 | - - 1. 153 | 2290 | 40 | 6 |
| - - 1. **F2** | - - 1. S01.217 | - - 1. 153 | 2004 | 21 | 12 |
